# The estimated impact of mandatory front-of-pack nutrition labelling policies on adult obesity prevalence and obesity-related mortality in England: a modelling study

**DOI:** 10.1016/j.lanepe.2025.101506

**Published:** 2025-11-04

**Authors:** Rebecca Evans, Martin O'Flaherty, I Gusti Ngurah Edi Putra, Chris Kypridemos, Eric Robinson, Zoé Colombet

**Affiliations:** a Department of Psychology, University of Liverpool, Liverpool, United Kingdom; b Department of Public Health, Policy and Systems, University of Liverpool, Liverpool, United Kingdom

**Keywords:** Microsimulation model, Policy evaluation, Inequalities, Food labelling policies, Obesity

## Abstract

**Background:**

Since 2013, front-of-pack traffic light labels have been implemented voluntarily on packaged food in the UK. The UK Government is now considering alternative labelling approaches which may be more effective, such as Chile's mandatory nutrient warning labels. The present study aimed to estimate the impact of mandatorily implementing (i) traffic light labels and (ii) nutrient warning labels on population-level obesity prevalence and mortality in England.

**Methods:**

A microsimulation model was built to simulate the effects of implementing mandatory front-of-pack nutrition labels (nutrient warning and traffic light) on assumed changes in daily energy intake from packaged food (due to consumer behaviour change and reformulation), and subsequent population-level obesity prevalence and mortality due to changes in BMI. We modelled the population of England, aged 30–89 years, over 20 years (2024–2043) using a synthetic population stratified by age, sex and Index of Multiple Deprivation. The model simulates individuals' life courses and counterfactuals under different policy scenarios, allowing detailed assessment of policies on exposures, disease risk, and mortality.

**Findings:**

Compared to the baseline scenario (current voluntary implementation of traffic light labelling), mandatory implementation of traffic light labelling was estimated to reduce obesity prevalence by 2.34 percentage points (95% UI 0.67–4.31) and prevent or postpone 57,000 (95% UI 13,000–160,000) obesity-related deaths. Mandatory implementation of nutrient warning labelling was estimated to have a larger impact; a 4.44 percentage point (95% UI 0.08–10.76) reduction in obesity prevalence and 110,000 (95% UI 2000–420,000) fewer obesity-related deaths.

**Interpretation:**

This work offers the first modelled estimation of the impact of introducing mandatory front-of-pack nutrition labels on obesity prevalence in the adult population in England. Findings suggest that mandatory implementation of nutrient warning labels would reduce prevalence of obesity and related deaths, compared to current voluntary or mandatory implementation of traffic light labelling, and should therefore be considered by the UK Government.

**Funding:**

European Research Council.

**Research in context**

**Evidence before this study**

We searched modelling studies on the impacts of nutrition labelling policies in PubMed from 1st January 2000 to 1st October 2024 using the search terms ((label polic∗ OR nutr∗ label∗ OR food∗ label∗ OR front of pack label∗ OR front-of-pack label∗ OR warning label∗ OR traffic light label∗) AND ((simulation OR microsimulation OR “model∗ study”)) based on titles and abstracts, restricted to human subjects. We identified 32 articles.

In the UK, a traffic light front-of-pack nutrition label has been implemented voluntarily since 2013 on approximately three-quarters of packaged foods. Yet, to date, no modelling studies have estimated the impacts of front-of-pack labelling on obesity prevalence in England. One study modelled the impact of nutrient warning labels in Mexico and estimated a 5% reduction in obesity 5 years post-implementation (4.98 [95% CI 3.93–6.03]; 1.3 million fewer cases). A handful of studies have modelled the impact of traffic light labelling on non-communicable disease (NCD) mortality. In Canada, it was estimated that 10,490 (95% CI 9312–11592) deaths per year would be prevented due to energy intake alone, if Canadians avoided foods labelled with red traffic lights. Another study estimated the impact of Nutri-Couleurs (traffic light label) across 27 EU nations and found no significant effect on NCD mortality.

**Added value of this study**

This study is the first to model the impact of front-of-pack labelling on obesity prevalence in England, and only the second to model the impact of nutrient warning labels on obesity prevalence. In the present study, a microsimulation model was built to estimate the impact of mandatorily implementing (i) traffic light labels and (ii) nutrient warning labels on obesity prevalence and deaths prevented or postponed via change in BMI. The UK Government is considering alternative front-of-pack labelling approaches, so findings can help to guide labelling policy development.

**Implications of all the available evidence**

Mandatory implementation of nutrient warning labels appears to be the most favourable policy option for the UK Government to reduce rates of obesity and related deaths, compared to current voluntary or mandatory implementation of traffic light labelling. Effects were equitable across different socioeconomic deprivation levels.

## Introduction

Diet-related disease is a major cause of poor population health and social inequalities in health.^1^ Many pre-prepared foods and non-alcoholic beverages (hereafter: food) are high in calories, added sugar, salt, and/or saturated fat.^2^^,^^3^ Excessive consumption of these nutrients increases the risk of obesity and other associated non-communicable disease (NCD) such as cardiovascular disease (CVD), and NCD mortality.^4^

In the UK, the average adult consumes an excess of 200–300 calories per day, and nearly two-thirds of UK adults are living with overweight or obesity.^5^^,^^6^ While energy intake alone does not capture diet quality, metabolic responses, or contextual factors that can moderate long-term weight gain, it remains a key factor contributing to obesity risk. Notably, the prevalence of overweight and obesity is patterned by deprivation (14 percentage points higher in the most deprived areas than in the least deprived areas) and education (12 percentage points higher for those with no qualifications compared to those who are degree-level educated).^5^ Therefore, there is a need for equitable public health policies that improve dietary quality across the population.

**Fig. 1:**
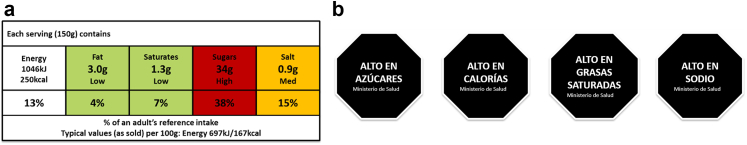
**Front-of-pack nutrition labels examples**. (a) Traffic light label (UK) redrawn.^8^ (b) Nutrient warning labels - black octagons (Chile) redrawn.^9^ In English, the nutrient warning labels would read (left to right): [HIGH IN] SUGAR, CALORIES, SATURATED FAT, SODIUM [Ministry of Health].

Front-of-pack nutrition labels are an evidence-based policy tool used to help consumers make healthier food choices and encourage industry to improve the nutritional profile of the products they sell.^7^ In the UK, a traffic light front-of-pack nutrition label (see Fig. 1A) has been implemented voluntarily since 2013. This traffic light label uses green, amber, and red colours to indicate whether a product contains low, moderate, or high levels of nutrients of concern (i.e., nutrients associated with increased NCD risk when consumed in excess), alongside guideline daily amount (GDA) percentages for each nutrient (typically per serving). However, UK consumers report that the traffic light label is difficult to interpret, which may widen health inequalities.^10^ Additionally, less than half of consumers use the label to determine product calorie content, likely because calorie content is not designated with a traffic light colour.^11^ It may be that simpler labels are required, as most consumers typically spend no more than a few seconds examining labels before making a food selection.^12^

In July 2020, the UK Government launched a consultation considering an alternative front-of-pack nutrition label to the traffic light.^13^ In the consultation, Chile's nutrient warning labels were highlighted as a potential alternative, and the benefits of implementing mandatory front-of-pack labelling were discussed. This was further discussed during a House of Lords inquiry on Food, Diet, and Obesity in 2024, in which there was a call for mandatory front-of-pack nutrition labelling and research into labelling systems that have demonstrated success in other countries, such as nutrient warning labels.^14^ Together, this suggests that the UK Government are actively considering nutrient warning labels as a front-of-pack labelling policy to address obesity.

In 2016, Chile implemented a mandatory policy requiring packaged foods containing ‘high’ amounts (as defined by thresholds set by the Ministry of Health) of calories, added sugar, sodium, and/or saturated fat to display nutrient warning labels^15^ (see Fig. 1B). Very similar policies have since been implemented in other Latin American countries.^16^^,^^17^ Mandatory nutrient warnings have also been implemented further afield in Canada and Israel, and policy development is under consideration in several other countries, including the US, India, and South Africa.^18^ Evidence indicates that implementation in Chile has reduced the purchase of energy (a relative 8.3% [95% CI 5.0–11.6] decrease) and nutrients of concern,^19^ and has led to product reformulation across all food groups, leading to reductions in energy content (−3.9%), and other labelled nutrients of concern.^20^

Furthermore, evidence from a meta-analysis of over 100 randomised controlled trials (RCTs) and quasi-experimental studies suggests that nutrient warning labels may perform better than traffic light labels in terms of reducing consumers’ purchase of energy (an additional 6.4% [95% CI 0.4–12.5] reduction) and nutrients of concern, and probability of choosing less healthy products.^7^ Therefore, it is important to examine the potential impact of their implementation in the UK on health outcomes such as adult obesity prevalence, to inform policy decision-making.

The present study aimed to estimate the likely long-term impacts of mandatorily implementing, in England, (i) traffic light labels and (ii) nutrient warning labels on packaged in-store foods, relative to the current voluntary implementation of traffic light labels, on energy intake and consequent population-level obesity prevalence and mortality via change in BMI.

## Methods

### Model overview

We built a dynamic, discrete-time, stochastic, open-cohort microsimulation model to quantify the estimated effects of implementing front-of-pack nutrition labels in England; an adaptation of the IMPACT NCD Model based on the IMPACT Food Policy Model.^21^ The model simulates the life-course of individuals and their counterfactuals under alternative policy scenarios. This enables the detailed simulation of diet policies and their impact on relevant exposures, subsequent disease epidemiology, and mortality, in a competing risk framework that accounts for different lag times between exposures and outcomes. In this case, we simulated the effects of implementing mandatory front-of-pack nutrition labels (traffic light and nutrient warning) on daily energy intake from packaged food, and subsequent population-level obesity prevalence and mortality due to changes in BMI. We modelled the population of England, aged 30–89 years, over 20 years (2024–2043) using a synthetic population stratified by age, sex and Index of Multiple Deprivation (IMD) that captures the observed demographics, energy intakes, and disease epidemiology of the actual population of England using available national data sources (see below and in Appendix section “Creation of our synthetic population”).

We evaluated two main policy scenarios:

1. Traffic light labels are implemented as a mandatory policy
2. Nutrient warning labels are implemented as a mandatory policy

We compared each scenario with a counterfactual “no intervention” (baseline) scenario, which corresponds to the current England legislation: continued voluntary implementation of traffic light labels.

We also modelled the impact of Nutri-Score, an alternative front-of-pack label which uses a colour spectrum and letter grades to summarise product healthiness, but not as a main scenario.^22^ This is because meta-analytic evidence suggests that it does not perform significantly differently to the traffic light label in terms of reducing energy purchased.^7^ Instead, results for Nutri-Score are presented in the Appendix (see Appendix Table S4).

### Front-of-pack nutrition labels

**Fig. 2:**
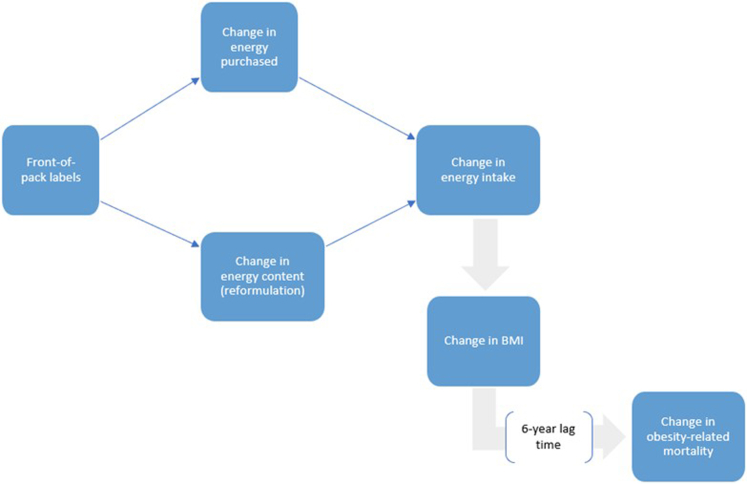
**Logic diagram of the impact of front-of-pack labelling on obesity prevalence and mortality**.

Front-of-pack nutrition labels impact diet through (i) consumer behaviour change, and (ii) industry response, i.e., reformulation of the products by industry (see Fig. 2).

### Effect on consumer behaviour change

We assumed that the traffic light labels and nutrient warning labels would reduce energy purchased from packaged food by 6.5% (95% CI 1.9–11.1), and 12.9% (95% CI 8.0–17.9) respectively, compared to no label, based on the estimates from Song et al.’s review and network meta-analysis.^7^ Based on the same meta-analysis, we assume that nutrient warning labels will outperform traffic light labels in reducing the total amount of energy purchased by 6.4% (95% CI 0.4–12.5]). Based on existing literature, we assumed no differential policy effects by sex, age or socioeconomic position.^7^^,^^23^ Due to an absence of evidence, we assumed both labels have a consistent effect on consumer behaviour over time. Specifically, we assumed that the labels would have an immediate effect on energy intake which remained stable at the new lower level across the modelled time horizon.

### Effect on energy content reformulation

For nutrient warning labels, we assumed a 3.9% (95% CI –12.5–5.0) reduction in energy content of labelled packaged foods, based on evidence from Chile post-implementation.^20^ While there is no available data specifically in relation to traffic light labelling and product reformulation, evidence suggests that a small amount of reformulation does occur in response to food labelling, particularly when it is implemented mandatorily.^24^, ^25^, ^26^ Therefore, we also assumed the same 3.9% reduction in energy content of packaged foods in response to mandatory traffic light labelling.

### Label coverage

We assumed that all packaged products (100%) would feature a traffic light label, as under mandatory implementation, this would be required by law.^19^ Under current voluntary implementation, it is estimated that 75% of packaged products feature the label,^27^ so mandatory implementation would yield an additional 25% coverage. Therefore, the consumer behaviour change and reformulation effects for mandatory traffic light labelling were applied to the estimated additional 25% packaged foods that would carry the label.

For nutrient warning labels, based on evidence on the proportion of products featuring a “high in” warning in Chile, we assumed that 51% (95% CI 49.0–52.0) of packaged foods in England would feature the label (i.e., will be above the phase 1 thresholds in Chile for warning).^28^ These thresholds closely align with the UK's red traffic light criteria for high nutrient of concern levels and are therefore the most relevant comparator for a potential implementation scenario (see Appendix Table S6 for detailed comparison).^28^^,^^29^ The nutritional quality of packaged food in Chile is relatively similar to the UK; the average Health Star Rating for packaged food is 2.44 in Chile compared to 2.83 in the UK (scores range from 0.5 to 5, with a higher score indicating better nutritional quality).^30^ Moreover, an analysis of food items from the UK NDNS indicated that approximately 40% of UK food items meet requirements for a red traffic light label, and this figure does not include items that would be labelled due to being high in energy.^31^ Research suggests that 32% of UK supermarket snack foods alone exceed adult energy intake recommendations^3^ and therefore it is reasonable to estimate that this would amount to at least an additional 10% of products being labelled, consistent with the 51% figure derived from Chile.

Based on this, the consumer behaviour change and reformulation effects for mandatory nutrient warning labelling were applied to the 51% of packaged foods estimated to carry the label. Effects from the meta-analysis reflect average changes in energy purchases across both labelled and unlabelled products, so accounting for label coverage avoids overestimation. This conservative approach may underestimate the true per-product effect but better reflects realistic implementation.

### Estimating model uncertainty

We used the Monte Carlo approach (200 iterations) to estimate the uncertainty of model parameters. This approach uses random sampling to create values for uncertain input values, taking into account the mean and lower and upper bounds of the confidence interval. The sources of uncertainty we considered were the uncertainty of the relative risk of coronary heart disease (CHD) mortality, stroke mortality and any other cause mortality based on BMI, the uncertainty of mortality forecasts, and the uncertainty of the policy (label) effect. We summarised the output distributions by reporting the medians and 95% uncertainty intervals (UIs).

### One-way sensitivity analyses

We performed one-way sensitivity analyses for label coverage, Chile's black octagon, and reformulation.

### Sensitivity analysis: change in nutrient warning labels coverage

Evidence from Chile suggests that approximately one year after the initial implementation of the nutrient warning label policy, reformulation resulted in a decrease in the proportion of products featuring a label from 51% to 44% (95% CI 42.0–45.0).^28^ Reformulation to reduce nutrients of concern is consistently observed in response to the introduction of front-of-pack nutrition labelling policies in various countries, including Australia, Canada, the Netherlands, and New Zealand, to avoid a “negative” label (e.g., a low health rating): or the absence of a “positive” label (e.g., a healthy choice indicator).^32^ Therefore, in this sensitivity analysis we assumed that coverage is 51% for the first-year post-implementation, and coverage then drops to 44% thereafter, while we left the reformulation effect unchanged.

### Sensitivity analysis: Chile's black octagon specifically (as opposed to nutrient warning labels more generally)

In this sensitivity analysis, we test based on evidence from Chile specifically, post-implementation (as opposed to meta-analytic data on nutrient warning labels in general from experimental studies), which suggests an overall 8.3% (95% CI 5.0–11.6) reduction in energy purchased.^19^ Notably, nutrient warning labels were introduced in Chile as part of a set of policies, including restrictions on food marketing to children, and therefore this reduction in energy purchase may not be wholly attributable to nutrient warning label implementation.

### Sensitivity analysis: consumer behaviour change + lower reformulation due to traffic light labels

It is possible that reformulation of energy content may be lower in response to traffic light labelling relative to nutrient warning labelling. This is because calories are not colour-coded in traffic light labels, and therefore, food companies may be less inclined to reformulate the energy content of products. We assumed there would be a smaller 0.9% (95% CI −3.1–4.9) reduction in energy content, based on a meta-analysis of food labelling effects on product energy reformulation.^26^

See Table 1 for a summary of key model assumptions. A further detailed description of the model, input sources, and key assumptions is provided in the Appendix.

**Table 1:** Summary of key model assumptions.

	Traffic light label	Nutrient warning label
*Main assumptions*		
Effect on energy intake via consumer behaviour change	−6.5% [−11.1; −1.9]^7^	−12.9% [−17.9; −8.0] (outperforms the traffic light label by 6.4% [0.4; 12.5])^7^
Effect on reformulation in terms of energy content	−3.9% [−12.5; 5.0]^20^	−3.9% [−12.5; 5.0]^20^
Label coverage on packaged products	100% (currently 75% under voluntary implementation)^27^	51.0% [49.0; 52.0]^28^
*Sensitivity assumptions*		
Changes in label coverage over time due to reformulation	–	Drops to 44.0% [42.0; 45.0] 4 years post-implementation^28^
Chile's black octagon nutrient warning label effectiveness on energy intake from labelled products	–	−8.3% [−11.6; −5.0]^19^
Effect on reformulation in terms of energy content	−0.9% [−3.1; 4.9]^26^	–

### Model engine

Front-of-pack nutrition labels are hypothesised to reduce energy intake, which will subsequently impact the body weight of the population (i.e., BMI), and, in turn, change mortality risk from CVD and non-CVD causes. This pathway is described in Fig. 2 and detailed in the Appendix (section “Estimating the effect of change in energy intake upon obesity prevalence and mortality”). In short, the change in energy intake is calculated by subtracting intake post-intervention from baseline intake for each year. Changes in energy intake are then converted into changes in body weight, based on principles of energy conservation, using the Christiansen & Garby prediction formula^33^ (detailed in Appendix section “Estimating the effect of change in energy intake on BMI”). The estimated change in BMI is then calculated based on the estimated change in body weight, which allows us to estimate the change in obesity prevalence. Next, these changes in BMI are used to estimate changes in mortality risk, with a 6-year lag time^34^ (see details in Appendix section “Estimating the effect of change in BMI upon mortality”). Using this information, new mortality rates and, consequently, the number of deaths projected can be estimated.

### Model outputs

The model produced the change in obesity prevalence and the total number of deaths prevented or postponed (DPPs) via a change in BMI for each scenario. Results are presented for adults in England aged 30–89 years, from 2024 to 2043, rounded to 2 significant figures for mortality and rounded to 2 decimal places for obesity prevalence.

### Data sources

We constructed a synthetic population of England to simulate the population-level impact of the policy scenarios. This is described in the Appendix section “Data sources used in our model” and Appendix Table S1. The England population projections were derived from the Office for National Statistics (ONS), and mortality trend projections were based on the deaths observed in England from 1981 to 2016.

We used generalised additive models for location, shape and scale (GAMLSS) to estimate (i) BMI and (ii) energy intake distributions dependent on year, age, sex, and IMD. GAMLSS can handle complex relationships between the response variable and its predictors and numerous types of distributions.^35^ Trends in energy intake, daily energy intake, and BMI were obtained from the nationally representative National Diet and Nutrition Survey (NDNS) 2009–2019. These trends in energy intake and BMI observed in the last 10 years in England were assumed to continue in the future. To obtain the daily energy from packaged food bought from grocery retail stores, we assumed that 55% of all food and beverage expenditure (including alcoholic beverages) was for at-home consumption (vs. 45% spent on restaurants and other out-of-home food services)^36^ and that 80% of the products purchased are packaged (vs. 20% fresh)^10^ (see details in Appendix section “Modelling approach and scenarios”).

R (version 4.3.0) was used to conduct all data management and statistical analyses. We used the “demography” package^37^ for forecasting mortality and the “gamlss” package to fit the distribution.^38^ For code, see https://github.com/zoecolombet/FoPLabels_code.

### Ethical approval

Ethical approval was not required for this study.

### Role of the funding source

The funder played no role in the study design, data collection, data analysis, data interpretation, writing of the paper, or the decision to submit this work for publication.

## Results

At the baseline scenario, maintaining current voluntary implementation of traffic light labelling in England was estimated to result in an obesity prevalence of 28.03% (95% UI 27.76–28.30) and approximately 16,000,000 (95% UI 8,200,000–30,000,000) deaths in English adults by 2043.

The implementation of mandatory traffic light labelling in England was estimated to reduce obesity prevalence by 2.34 percentage points (95% UI 0.67–4.31), of which 0.82 percentage points (95% UI 0.00–2.70) could be attributed to reformulation. Implementing mandatory nutrient warning labels was estimated to have a larger impact and reduce obesity prevalence by 4.44 percentage points (95% UI 0.08–10.76), of which 1.42 percentage points (95% UI 0.00–7.09) could be attributed to reformulation. See Table 2.

**Table 2:** Estimated change in obesity prevalence and mortality due to change in BMI in adults in England (2024–43), according to different front-of-pack labelling implementation scenarios.

	Change in prevalence of obesity (percentage points)	Obesity-related deaths prevented or postponed
**Consumer behaviour change**		
Traffic light labelling (mandatory)	−1.42 (−2.45; −0.42)	26,000 (5500; 72,000)
Nutrient warning labelling (mandatory)	−2.64 (−7.70; 0.00)	46,000 (0; 220,000)
**Reformulation**		
Traffic light labelling (mandatory)	−0.82 (−2.70; 0.00)	14,000 (0; 69,000)
Nutrient warning labelling (mandatory)	−1.42 (−7.09; 0.00)	24,000 (0; 200,000)
**Combined**		
Traffic light labelling (mandatory)	−2.34 (−4.31; −0.67)	57,000 (13,000; 160,000)
Nutrient warning labelling (mandatory)	−4.44 (−10.76; −0.08)	110,000 (2000; 420,000)

Implementing traffic light labelling mandatorily was estimated to result in approximately 57,000 (95% UI 13,000–160,000) obesity-related DPPs, of which 14,000 (95% UI 0–69,000) could be attributed to reformulation. Again, implementing mandatory nutrient warning labels was estimated to have a larger impact, resulting in an estimated 110,000 (95% UI 2000–420,000) obesity-related DPPs, of which 24,000 (95% UI 0–200,000) could be attributed to reformulation. See Table 2. Overall, mandatory implementation of nutrient warning labels was estimated to have a larger combined impact than mandatory traffic light labelling on both obesity prevalence (in 77% of simulations) and obesity-related DPPs (in 76% of simulations).

At the baseline scenario, maintaining current voluntary implementation of traffic light labelling in England was estimated to result in an obesity prevalence of 32.52% (95% UI 31.97–33.11) and approximately 3,500,000 (95% UI 1,800,000–6,400,000) deaths in the most deprived adults by 2043, and an obesity prevalence of 24.26% (95% UI 23.58–24.90) and approximately 2,900,000 (95% UI 1,400,000–5,500,000) deaths in the least deprived.

The introduction of either front-of-package label as a mandatory policy is estimated to reduce obesity prevalence and related deaths to a similar extent across socioeconomic deprivation levels (see Table 3). Based on our modelling results, the impact of either of these policies on socioeconomic inequalities in health is highly uncertain, however it is more likely than not for these policies to reduce existing health inequalities.

**Table 3:** Estimated change in obesity prevalence and mortality due to change in BMI in adults in England (2024–43), according to IMD quintile groups and different front-of-pack labelling implementation scenarios.

	Estimated change in obesity prevalence, percentage points	Estimated obesity-related deaths prevented or postponed	Deaths prevented or postponed per capita ratio (*Q1 [most deprived]/Q5 [least deprived]*)^a^	Probability of equitable policy^b^
**Mandatory traffic light labelling**—**consumer behaviour change**				
*Q1 (most deprived)*	−1.36 (−2.28; −0.41)	5500 (500; 16,000)	1.15	0.55
*Q5 (least deprived)*	−1.43 (−2.39; −0.40)	4500 (500; 13,000)		
**Mandatory traffic light labelling–reformulation**				
*Q1 (most deprived)*	−0.75 (−2.65; 0.00)	3000 (0; 15,000)	1.26	0.48
*Q5 (least deprived)*	−0.87 (−2.58; 0.00)	2500 (0; 12,000)		
**Mandatory traffic light labelling–combined**				
*Q1 (most deprived)*	−2.21 (−4.12; −0.67)	11,000 (2000; 35,000)	1.11	0.59
*Q5 (least deprived)*	−2.32 (−4.33; −0.67)	10,000 (2000; 28,000)		
**Mandatory nutrient warning labelling—consumer behaviour change**				
*Q1 (most deprived)*	−2.55 (−7.60; 0.00)	9500 (0; 48,000)	1.19	0.62
*Q5 (least deprived)*	−2.65 (−7.79; 0.00)	8000 (0; 38,000)		
**Mandatory nutrient warning labelling–reformulation**				
*Q1 (most deprived)*	−1.37 (−6.87; 0.00)	5000 (0; 46,000)	1.21	0.50
*Q5 (least deprived)*	−1.50 (−7.14; 0.00)	4500 (0; 29,000)		
**Mandatory nutrient warning labelling–combined**				
*Q1 (most deprived)*	−4.26 (−10.55; −0.09)	21,000 (0; 88,000)	1.10	0.63
*Q5 (least deprived)*	−4.49 (−10.47; −0.09)	20,000 (0; 78,000)		

a Medians of ratios of DPP rates (per capita) between most and least deprived IMD groups; Values above 1 denote that the most deprived quintile group might benefit more as a result of the policy, compared to the least deprived quintile group.

b The probability that the policy might be equitable, i.e., it is more effective in the most deprived quintile group compared to the least deprived. Values above 0.5 denote that the policy is more likely than not to prevent more deaths per capita in the most deprived quantile group.

See Appendix Table S3 for sensitivity analysis results relating to nutrient warning label coverage, Chile's nutrient warning label specifically, and traffic light label reformulation. Briefly, nutrient warning labels with reduced coverage, and Chile's warning label specifically still outperformed traffic light labels. Traffic light labels saw a decrease in performance using the more conservative reformulation estimate (combined impact lower than nutrient warning labels for both obesity prevalence [in 84% of simulations] and obesity-related DPPs [in 84% of simulations]). See Appendix Table S4 for results relating to Nutri Score. As expected, results for Nutri Score were very similar to those for traffic light labelling. See Appendix Table S5 for results on obesity-related CVD mortality specifically.

## Discussion

This study offers the first modelled estimation of the impact of changing front-of-pack nutrition label policy on obesity prevalence and mortality in the adult population in England. Our findings indicate that, in place of current voluntary traffic light labelling, the introduction of mandatory nutrient warning labels would reduce obesity prevalence and related deaths substantially more than making traffic light labels mandatory, with no differential effects on health inequalities.

Our findings are largely consistent with the existing limited modelling evidence in this area. One previous study modelled the impact of nutrient warning labels in Mexico.^39^ The study estimated a mean caloric reduction of 36.8 kcal/day/person, and, 5 years post-implementation, 1.3 million fewer cases of obesity (5% reduction). A handful of studies have modelled the impact of traffic light labelling on NCD mortality. One study modelling the impact in Canada^40^ estimated that 10,490 deaths per year due to energy intake alone would be prevented. However, this was contingent on Canadians using the traffic light labelling to avoid foods labelled with red traffic lights. Another study estimated the impact of Nutri-Couleurs (traffic light label) across 27 EU nations and found no significant effect on NCD mortality.^41^ Notably, the effect estimate for change in energy intake was derived from a large-scale randomised controlled trial in French supermarkets, which only covered four product types (bread, ready meals, fresh catering, and pastries).^42^

Although the current research provides important insights into the likely impact of changing front-of-pack nutrition label policy in England, there are limitations to be acknowledged. We assumed that reductions in energy intake would be in response to labelled products, which may be an overestimate for traffic light labels, as not all labelled products would be high in energy or feature a “red” nutrient indicator. Additionally, the effects of labels on intake were largely derived from studies in controlled settings and may not reflect actual grocery shopping behaviours in response to labels. Specifically, actual grocery shopping involves competing factors such as a wider product offering, marketing strategies (e.g., price promotion), and actual financial cost. We also assumed that energy intake trends from NDNS will continue, but it is possible that COVID-19 and/or the cost-of-living crisis may result in long-term changes. We assumed that food purchase would directly impact food intake. Food waste may lead to discrepancies between what is purchased and what is ultimately consumed. However, packaged foods such as confectionery and snacks are less commonly wasted than fresh produce.^43^ We also assumed that purchases at the household level would be evenly distributed across individuals in that household. It is likely that actual intake would vary based on demographic factors, such as age and sex; however, this bias should be mitigated when comparing scenarios. Our results will also underestimate total policy benefits, as we did not include children and young adults (<30 years) in our model, because although obesity prevalence is high in younger age groups, obesity/CVD mortality is rare, so validated risk assessments are limited.

It is also important to acknowledge that the present research underestimates the impact of the labelling policies on mortality, as due to model design, we do not model effects of policies due to changes in intake of nutrients of concern (salt, sugar, saturated fat) and instead model change via energy intake and reductions to BMI. Excess intake of salt, sugar, and saturated fat is associated with CVD risk.^44^ Evidence suggests that labelling policies decrease the purchase of nutrients of concern, especially nutrient warning labels relative to traffic light labels, so impacts on CVD mortality are likely to be particularly underestimated for nutrient warning labels.^7^^,^^23^ Policy impact for nutrient warning labels may also be underestimated due to the conservative approach of only applying purchase effects to labelled pre-packaged products, rather than both labelled and unlabeled products. Moreover, while energy intake is a key factor contributing to changes in BMI, it is only a partial determinant of obesity risk. Obesity arises from a complex interplay of biological, behavioural, and environmental factors; energy intake alone does not capture diet quality, metabolic responses, or contextual drivers that can moderate long-term weight gain.

We did not model a scenario where nutrient warning labels are implemented voluntarily, as we assume it is very unlikely that the food industry would agree to this. Moreover, the success of voluntary, industry-endorsed initiatives is largely contingent upon industry uptake. Current evidence also suggests that voluntary implementation may also be less effective for several reasons, such as industry manipulation of label design, noncompliance (particularly as nutrient warning labels are known to deter purchase of labelled products), and a lack of independent target setting, monitoring, and enforcement.^45^^,^^46^ While data from the most comprehensive meta-analysis to date was used to model labelling effects on consumer behaviour, effects were derived from a handful of predominantly laboratory-based studies outside the UK. Additional real-world studies with UK consumers are needed to improve confidence in the modelled effects and subsequent estimates. Moreover, several assumptions in our model were constrained by a lack of available evidence, and these areas might benefit from further research. Firstly, there was no available data on how the effect of the label on consumer behaviour change may change over time. Theoretically, if people become habituated to front of pack labels, then the effect may decrease, or conversely, if nutrient literacy and awareness strengthen over time, then the effect may increase.^10^ This novelty-familiarity trade-off is important to consider as UK consumers are habituated to traffic light labels (more than 90% recognise them^11^). Secondly, there was no available data on compensatory effects from the intake of fresh food in place of packaged food, or intake from out-of-home eating. Thirdly, although there is some self-report evidence to suggest that age, education, and ethnicity may impact understanding of, and therefore response to traffic light labels,^10^ there was no consistent evidence that demographic factors moderate the effect of labels on product choice.^7^^,^^23^ Finally, there was limited data available for reformulation in response to specific front-of-pack labelling approaches. Label coverage and reformulation estimates for nutrient warning labels were derived from Chile. While evidence suggests that the nutritional quality of the packaged food offering in the UK and Chile is similar, this does not necessarily mean that the proportion of products requiring a label or reformulation in response to labelling would be comparable.^28^^,^^30^^,^^31^ Moreover, existing UK policies, such as sugar and calorie reduction targets and the Soft Drinks Industry levy, are likely to moderate reformulation effects over the modelled horizon.

The World Health Organization (WHO) does not at present recommend the use of any specific labelling scheme but encourages research institutions and member states to continue analysing information to inform decisions.^47^ Nutrient warning labels are gaining global popularity, but the UK and the rest of Europe are yet to adopt this policy approach. While there may be political, regulatory, and cultural reasons why nutrient warning labels have not been adopted in Europe, notably, the UK is no longer a member of the EU and because of this is not required to harmonise any mandatory front-of-pack labelling approach with other EU member states. This new modelled evidence supports the use of nutrient warning labels to reduce population-level obesity. However, it is important to acknowledge the limitations of the present work; we were constrained by a lack of evidence in the UK and wider European Region, and therefore further country-specific evidence is required to improve confidence in policy impact estimates.

Based on our modelled evidence, mandatory implementation of nutrient warning labels appears to be the most favourable policy option for the UK Government to reduce rates of obesity and obesity-related mortality, compared to current voluntary or mandatory implementation of traffic light labelling. Further real-world evidence with UK consumers is required to improve confidence in policy impact estimates.

## Contributors

ZC, RE, ER, MO'F, and IGNEP designed the study. ZC, RE, and CK directly accessed and verified the underlying data reported in this article. ZC, RE, and CK developed the model. CK, MO'F, and ER supervised ZC and RE. RE and ZC did the analysis and drafted the manuscript. All authors contributed to the data interpretation and revised each draft for important intellectual content. All authors had final responsibility for the decision to submit for publication.

## Data sharing statement

ONS and NDNS data are available online. The “demography” package for R software has been used for forecasting mortality and the “gamlss” package has been used to fit the distribution. Syntax for the generation of derived variables and for the analysis used in this study are available publicly: https://github.com/zoecolombet/FoPLabels_code.

## Declaration of interests

IGNEP declares funding from the National Institute of Health and Care Research (NIHR) Development and Skill Enhancement Award (DSE), outside of this work. All other authors declare no competing interests.
